# Alzheimer’s disease as a chronic maladaptive polyamine stress response

**DOI:** 10.18632/aging.202928

**Published:** 2021-04-03

**Authors:** Baruh Polis, David Karasik, Abraham O. Samson

**Affiliations:** 1 Drug Discovery Laboratory, The Azrieli Faculty of Medicine, Bar-Ilan University, Safed 1311502, Israel; 2 Hebrew SeniorLife, Hinda and Arthur Marcus Institute for Aging Research, Boston, MA 02131, USA; 3 Musculoskeletal Genetics Laboratory, The Azrieli Faculty of Medicine, Bar-Ilan University, Safed 1311502, Israel

**Keywords:** polyamines, aging, senescence, arginase, neurodegeneration

## Abstract

Polyamines are nitrogen-rich polycationic ubiquitous bioactive molecules with diverse evolutionary-conserved functions. Their activity interferes with numerous genes' expression resulting in cell proliferation and signaling modulation.

The intracellular levels of polyamines are precisely controlled by an evolutionary-conserved machinery. Their transient synthesis is induced by heat stress, radiation, and other traumatic stimuli in a process termed the polyamine stress response (PSR).

Notably, polyamine levels decline gradually with age; and external supplementation improves lifespan in model organisms. This corresponds to cytoprotective and reactive oxygen species scavenging properties of polyamines. Paradoxically, age-associated neurodegenerative disorders are characterized by upsurge in polyamines levels, indicating polyamine pleiotropic, adaptive, and pathogenic roles. Specifically, arginase overactivation and arginine brain deprivation have been shown to play an important role in Alzheimer’s disease (AD) pathogenesis.

Here, we assert that a universal short-term PSR associated with acute stimuli is beneficial for survival. However, it becomes detrimental and maladaptive following chronic noxious stimuli, especially in an aging organism. Furthermore, we regard cellular senescence as an adaptive response to stress and suggest that PSR plays a central role in age-related neurodegenerative diseases' pathogenesis.

Our perspective on AD proposes an inclusive reassessment of the causal relationships between the classical hallmarks and clinical manifestation. Consequently, we offer a novel treatment strategy predicated upon this view and suggest fine-tuning of arginase activity with natural inhibitors to preclude or halt the development of AD-related dementia.

## INTRODUCTION

Throughout their existence, organisms are repeatedly exposed to various stresses and thus have evolved species-specific strategies to effectively resist them and improve survival. Drought, starvation, heat, and cold shocks are among the universal stressful stimuli, which living creatures encounter during their life cycle. In addition, the organisms utilizing aerobic respiration or photosynthesis to produce energy continually deal with numerous oxidative challenges related to reactive oxygen and nitrogen species. Remarkably, similar to starvation and heat shock, oxidative stress induces polyamine synthesis in various species [1].

Polyamines are omnipresent primordial polycationic bioactive molecules possessing multifarious evolutionary-conserved biochemical functions. They are found ubiquitously at much higher concentrations than most other cellular metabolites [2]. The polyamine, spermidine, was presumably present in the last universal common ancestor (LUCA). Other polyamines are essential for growth and biofilm formation in simple bacteria [3]. Some bacteria produce diamines as a response to acid stress, via decarboxylation of arginine, ornithine, and lysine [4]. In plants, polyamines are omnipresent and protective in stressful events such as sudden drought and extreme salinization [5].

**Figure 1:**
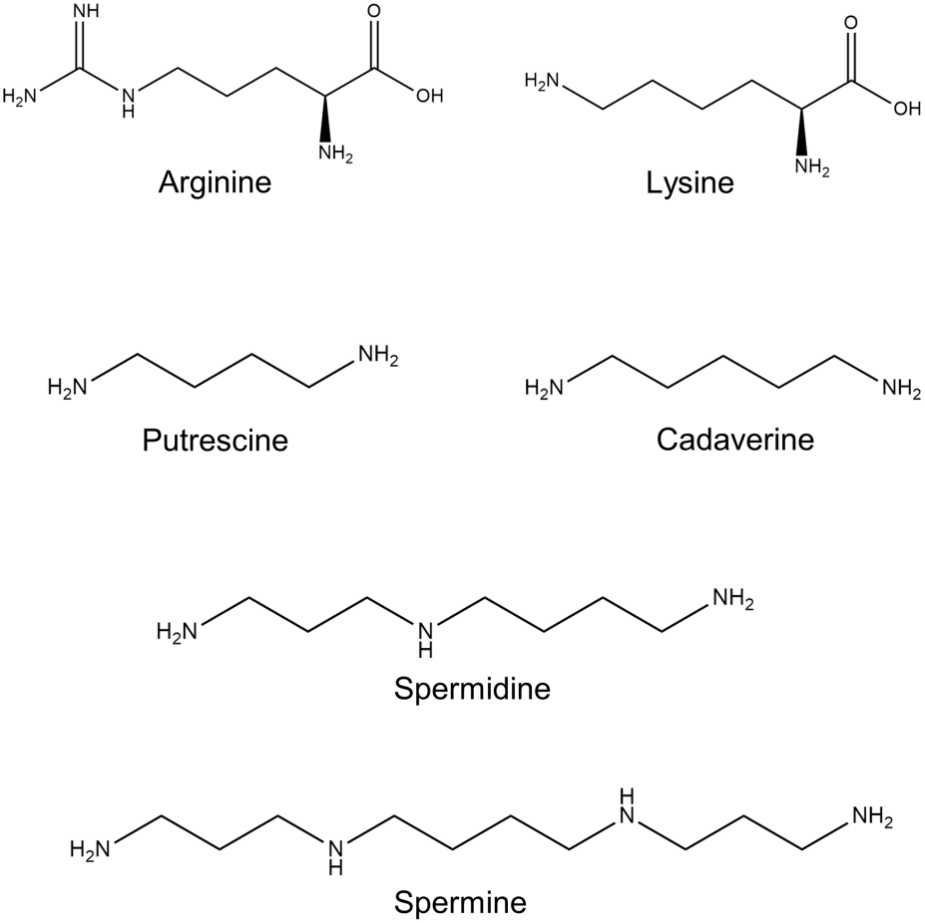
**Polyamine chemical structure.** Shown are the chemical structures of the polyamine precursors amino acids L-arginine and L-lysine; and polyamines: putrescine, spermidine, spermine, and cadaverine.

In mammals, polyamines are universal regulators of basal cellular functions. The natural polyamines spermidine and spermine, their diamine precursor putrescine, together with diamine cadaverine, are aliphatic molecules (Figure 1). They carry several nitrogen moieties, which are positively charged under physiological conditions. This feature provides them with an ability to interact with negatively charged nucleic acids and various proteins and influence critical cell functions via regulation of transcription, translation, and posttranslational modifications of a wide range of genes and proteins.

The levels and activity of enzymes involved in polyamines’ biosynthesis and metabolism are regulated by various specialized and unconventional mechanisms at numerous levels. Their transcription, translation, posttranslational modification, and finally, degradation processes, comprise complex feedback mechanisms monitoring the substrates and products concentrations [6]. These mechanisms are highly conserved in different species, which points to a central role of polyamines’ function in all known life forms. Even though polyamines are universal primordial life elements, substantial gaps in the understanding of their precise physiological roles still exist. Moreover, polyamines’ role in the pathogenesis of neurodegenerative diseases has received particularly scant scientific attention.

Recent discoveries have revealed many indispensable polyamines’ biological functions and attracted weighty attention to their evolutionary role in health and disease. In adult organisms, numerous stimuli have been shown to alter polyamine homeostasis and elicit a highly concerted polyamine stress response (PSR) [7]. Accruing evidence suggests that these alterations are adaptive and beneficial when they follow a moderate temporary stimulus, while tenacious stress leads to a maladaptive polyamine response, which contributes to malfunction and, eventually, degeneration. This maladaptive response characterizes pathogenesis of neurodegenerative disorders with typical arginase upregulation, arginine brain deprivation, and substantial increase in the brain levels of polyamines [8–11].

Consequently, we postulate that polyamines function as universal bivalent regulators of cellular functions, which either promote growth of cells or induce their death depending on environmental signals. This view is in line with the recent hypotheses suggesting that natural polyamines are beneficial for the physiological processes in healthy cells, but excessively detrimental under some pathological conditions [12] hence activating senescence as an adaptive response to stress [13, 14]. Accordingly, we regard cell senescence as a complex stress response phenotype contributing to neurodegeneration and progressive neuronal loss [15, 16].

Remarkably, polyamines-related adaptive response share some similarities with the prokaryotic stringent response and the eukaryotic unfolded protein response (UPR) [1]. The former is a ubiquitous bacteria and chloroplast (plant organelle) stress response to starvation, heat shock, and other stimuli. The latter is related to the endoplasmic reticulum (ER) stress and is a common response within yeast, worms, and mammals. Therefore, PSR is one of the earliest mechanisms that, along with other mechanisms, cope with stresses and improve survival.

Gilad and Gilad (2003) have proposed a model where the brain PSR is a component of the coordinated cellular stress program [17]. Here, we further develop this hypothesis and propose an original model that throws light on the pathogenesis of age-associated neurodegenerative diseases, and of Alzheimer’s disease (AD), in particular. We also describe a novel treatment strategy predicated upon this view and suggest fine-tuning of arginase activity with natural inhibitors to preclude or halt the development of clinical dementia, and perhaps even delay aging.

## Biosynthesis and catabolism of polyamines

Polyamine metabolism has been reviewed in detail by several authors. The interested reader is referred to an excellent review by Wallace et al*.* [18]. Here, we cover a topic that has received a relatively limited scientific attention and underline the role of enzymes that have been shown to play a role in the pathogenesis of neurodegenerative disorders.

**Figure 2:**
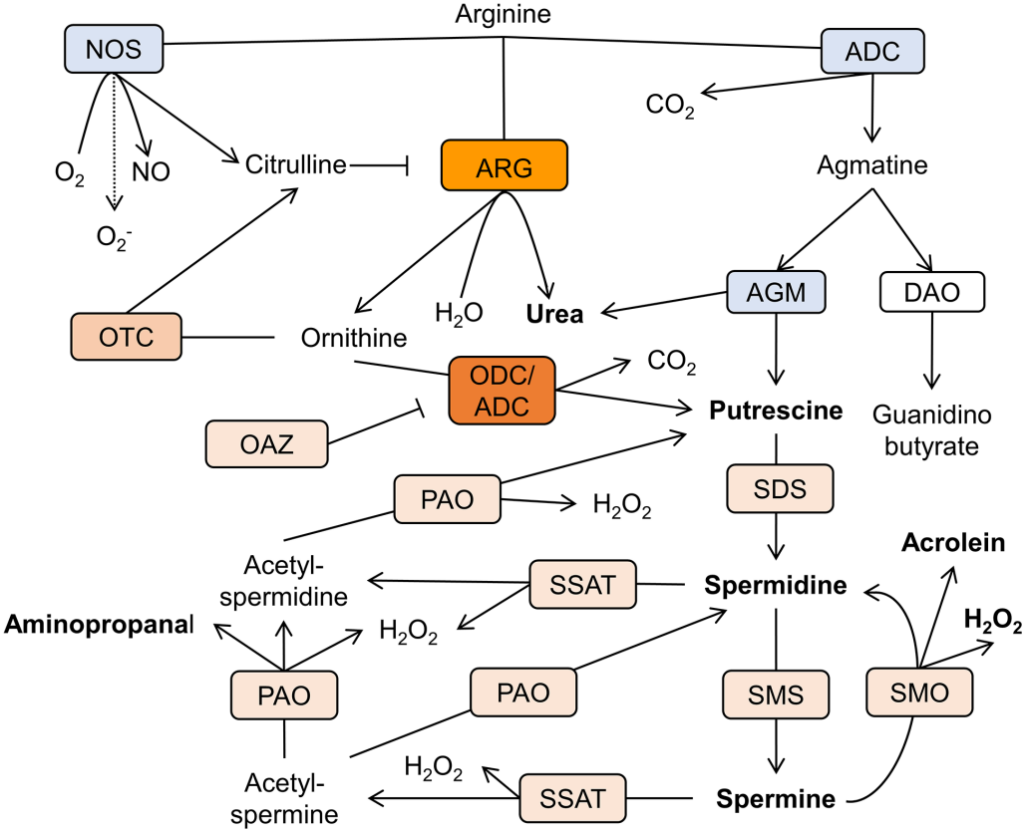
**Schematic representation of the polyamine metabolism pathways in AD brain.** Arginine is the mutual substrate for arginase (ARG), arginine decarboxylase (ADC), and nitric oxide synthase (NOS). Ornithine decarboxylase (ODC) decarboxylases ornithine to produce putrescine. Spermidine/spermine acetyltransferase (SSAT) catalyzes the acetyl-group transfer from acetyl-coenzyme A to the aminopropyl end of spermidine or spermine, producing acetylspermidine and acetylspermine. Acetylated polyamines are oxidized by polyamine oxidase (PAO) to produce hydrogen peroxide (H2O2), aminopropanal, and either putrescine or spermidine. Otherwise, spermine can be directly oxidized to spermidine by spermine oxidase (SMO) generating H2O2 and aminopropanal, which is spontaneously converted to acrolein. Other abbreviations: ornithine transcarbamylase (OTC), agmatinase (AGM), ODC antizyme (OAZ), spermidine synthase (SDS), spermine synthase (SMS), diamine oxidase (DAO). Rectangles’ color reflects the level of enzymes’ expression in relation to the healthy brain. Blue- reduction, shades of orange- increase in levels (arbitrary scale).

The cellular levels of polyamines in various tissues are congruous with their physiological requirements. This is achieved by a joint function of synthesis, catabolism, and transport. The chief biosynthetic polyamines' pathway in mammals utilizes arginine as a precursor of putrescine and comprises arginase and ornithine decarboxylase (ODC) or, alternatively, arginine decarboxylase (ADC), and agmatinase (AGM) [19] (Figure 2).

In all life forms, putrescine is the most common diamine. Most eukaryotes produce putrescine via decarboxylation of ornithine. The polyamine, cadaverine, a product of lysine decarboxylation, is much less abundant in mammal species. Cadaverine is a precursor of piperidine, which efficiently accumulates against concentration gradient in the murine brain [20]. Significant amounts of cadaverine are formed and resorbed in the intestine; however, endogenous sources of cadaverine in the mouse brain had been evidenced too [21] and suggested to be responsible for some central nervous system (CNS) functions [22]. In addition, cadaverine can be utilized by the gut *Escherichia coli* in an alternative pathway to produce putrescine [23].

**Figure 3:**
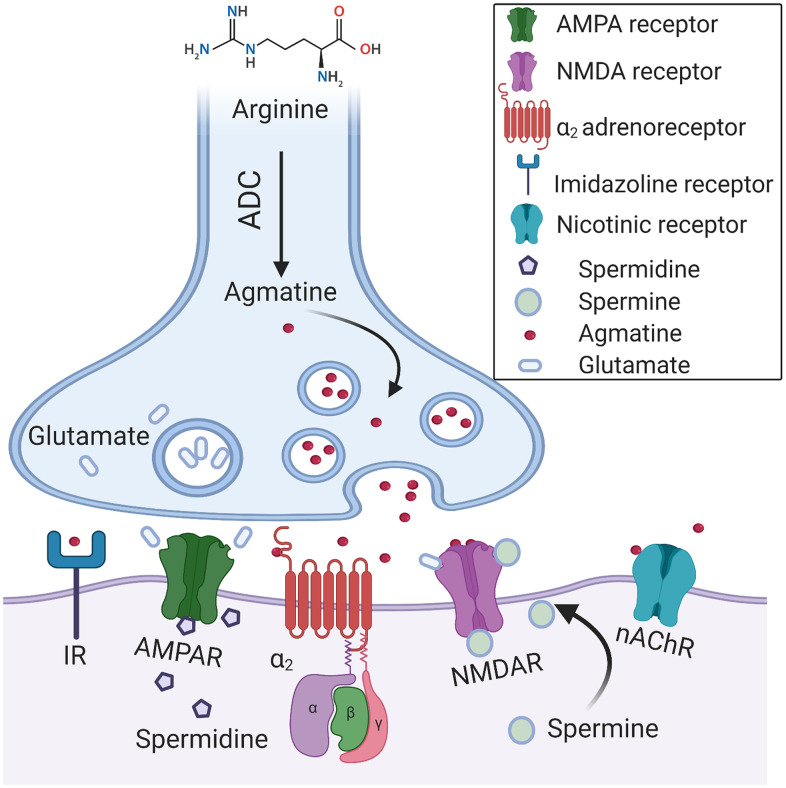
**A hypothetical synapse diagram.** Arginine decarboxylase (ADC) converts arginine into agmatine and carbon dioxide. Agmatine is a neurotransmitter that is synthesized, stored in vesicles, and released following depolarization. Agmatine binds with high affinity to α2-adrenoceptors (α2) and imidazoline receptors (IR). It antagonizes glutamatergic NMDA, AMPA, and nACh receptors. Polyamines, spermine, and spermidine modulate the activation of NMDA receptors via a unique allosteric regulatory site at the extracellular domain. The pore of ionotropic glutamate receptors is easily accessible to cationic polyamines, which are capable of blocking the ions movement via the channels. Polyamines efficiently block ACh-induced currents via nAChR.

In accordance with the dominant view, ODC is the limiting factor of the polyamines’ biosynthesis in mammals [6]. However, recent attention has been turned to arginase and ADC as putative gate keepers of polyamine synthesis. Agmatine has been recognized long ago as an ADC product in primitive life forms; nevertheless, ADC expression in mammals was doubted. Li et al*.* (1994) investigated the bovine brain and provided conclusive evidence that agmatine is an endogenous imidazoline receptors agonist. The authors demonstrated that agmatine is a locally synthesized noncatecholamine ligand of α2-adrenergic receptors that acts as a neurotransmitter [24] (Figure 3). Of note, both glia and mature neurons demonstrate ADC activity [25]. ADC activity has been identified in other organs and various cell types [26]. Several groups recurrently demonstrated the presence of mammalian ADC in rodents and humans [25, 27]. Moreover, it has been proven that ADC gene is responsible for the production of agmatine in the brain [25]. Additionally, it was revealed that ADC is associated with mitochondrial membranes and capable of decarboxylating both arginine and ornithine [28]. Remarkably, the brain ADC possesses a higher affinity for ornithine than for arginine [27] (Figure 2). Accordingly, in the cases of the mutual substrate (arginine) deficiency and relative ornithine excess, due to arginase upregulation for instance, ADC acts together with ODC to produce putrescine, which leads to a substantial decrease in the brain agmatine content.

Of note, agmatine demonstrates a broad spectrum of pharmacological actions in mammals, which include anti-nociceptive [29] and anti-inflammatory effects [30]. Likewise, it protects neurons against glutamate-induced cytotoxicity *in vitro* [31] and ischemic neuronal injury *in vivo* [32] (Figure 3). Therefore, its deficit may have serious effects on brain function, particularly in the conditions of oxidative stress.

Curiously, despite extensive literature, there are still papers suggesting that agmatine is synthesized exclusively by plants and bacteria, but not by mammals [12]. This proposition is indefensible. Moreover, mammalian ADC is unique and substantially distinct from ODC and ADC enzyme of bacteria and plants [27]. In prominent opposition to other polyamine pathway enzymes, ADC and arginase are both constitutively active enzymes, which are indispensable for polyamine synthesis, and are extensively expressed in the mammal brain. The cortical and hippocampal principal cells and interneurons clearly express both of them [33].

## Arginase

Remarkably, the minimal enzymatic content of the LUCA already included representatives of the arginase superfamily [34], pointing to their significance for the perpetuation of life. Arginase is a particularly interesting enzyme that has been present in early life forms and conserved throughout evolution. It is a manganese metalloenzyme catalyzing the hydrolysis of arginine to ornithine and urea in the last step of the urea cycle [35] (Figure 2). Two distinct genetic isoforms of human arginase, arginase-1 (Arg1) and arginase-2 (Arg2), share 59.4% of amino acids sequence [33, 36]. Structurally, both isoenzymes are homotrimers stabilized in conformation by two Mn^2+^ ions per each monomer [37].

Of note, the duplication of the arginase coding gene is a relatively recent evolutionary event, which occurred following the separation of vertebrates and invertebrates [38]. Simple organisms, such as plants, bacteria, and yeasts, possess a single form of Arg2 situated in the mitochondria. Mitochondrial localization of Arg2 indicates its evolutionary roots in bacteria [39]. Vertebrates additionally express a cytosolic isoform, Arg1 [35]. Therefore, it is presumed that the mitochondrial arginase is the ancestral isoform [40].

In mammals, both arginase isoforms show distinctive cell and tissue distribution patterns. They are encoded by genes in separate chromosomes; however, their mechanism of action and products are similar. Arg1 is generally presented as a cytosolic enzyme of the liver and is very common in other tissues including the brain. Arg2, in contrast, is described as a kidney-type mitochonrial enzyme; however, its expression has been shown in the mitochondria of various organs including the brain tissue [11, 41].

The chief function of arginase in ureotelic animals, being the last enzyme of the urea cycle, is to deal with excess of ammonia [42] (Figure 2). However, recent discoveries indicate the enzyme’s role in diverse physiological functions and pathological processes that are far beyond the urea cycle. The presence of both isoforms in the murine brain tissue, and particularly in the hippocampal neurons, has been proven by several groups [11, 43]. There are estimations that arginase brain activity is equally accounted for by both isoforms [40, 44], though Arg2 has been shown to be a dominant isoenzyme in the human frontal cortex [45].

Of importance, the levels of two central urea cycle enzymes, namely ornithine transcarbamylase (OTC) and carbamoyl phosphate synthetase 1 (CPS1), in the healthy mammal brain are extremely low [46], which points to a unique function of arginase in the CNS.

Typically, the expression of arginase is inducible by a variety of cytokines and catecholamines. It has been shown that 5′ flanking region of Arg1 gene contains elements responsive to interleukin-4, cAMP, tumor growth factor β, dexamethasone, and lipopolysaccharides (LPS) [47]. Oxidative species also instigate Arg1 expression and stimulate its activity via Protein kinase C-mediated activation of RhoA/Rho kinase pathway [48].

**Figure 4:**
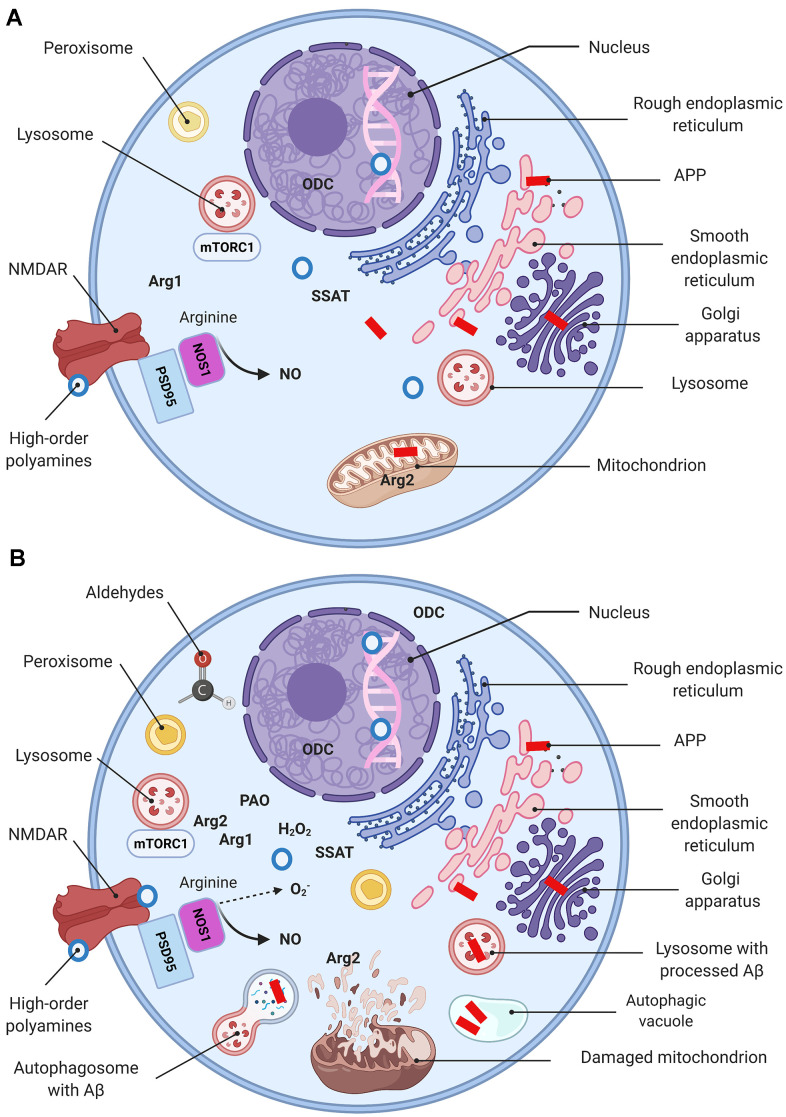
**Molecular basis of AD (a simplified model).** Schematic representation of a “normal” (**A**) and a dysfunctional (**B**) neuron with main organelles involved in APP processing and PSR. Following synthesis, APP undergoes posttranslational modifications in the ER and Golgi where it generates Aβ. In the healthy brain Aβ is localized in perikarya, trans-Golgi network, Golgi-derived vesicles (**A**), while oxidative stress upsurges intralysosomal Aβ content (**B**) (149). Mitochondria are essential for maintaining neuronal integrity and function. Arg2 is a typical mitochondria-associated enzyme (**A**). Mitochondria are targets for Aβ/ROS-mediated damage, which leads to swelling, outer membrane rupture, and followed by Arg2 appearance in the cytoplasm. Arg2, in turn, induces re-distribution of lysosome and mTOR from perinuclear area to cell periphery (**B**), activates mTORC1-S6K1 signaling and contributes to cell senescence phenotype characterized by impaired autophagy and apoptosis. This process eventuates in impediment of the autophagolysosomes maturation and lysosome-associated Aβ degradation, and leads to Aβ accumulation in autophagic vacuoles (**B**). Peroxisomes are not competent to cope with growing oxidative stress and become target for ROS. Oxidative stress leads to Arg1 overexpression, ODC translocation to the cytoplasm, and eventuates by polyamine overproduction (PSR). High-order polyamines stabilize the DNA conformation and modulate the chromatin structure and gene transcription via ionic interactions (**A**). Their elevated levels shut down some vital genes (**B**). Extensive polyamine catabolism is followed by the generation of hydrogen peroxide and cytotoxic aldehydes. NOS1 deprived of arginine undergoes uncoupling and switches to the production of superoxide anion. NMDA receptor function is modulated by polyamines via a special recognition site.

Arg2 levels in endothelial cells escalate as a reaction to numerous stimuli as well, including bacterial LPS, tumor necrosis factor alpha (TNFα), oxidized low-density lipoproteins (LDL), and hypoxia [49]. Remarkably, Arg2 activation in the endothelial cells is associated with its translocation from the mitochondria to the cytosol [50]. A similar pattern has been reported in the hippocampal neurons of AD mice [11]. It is well-established that AD-associated conditions with elevated ROS result in mitochondrial swelling, outer membrane rupture, and the cell death induction [51]. Therefore, the presence of Arg2 in the cytoplasm may be explained by the disease-associated severe mitochondrial damage (Figure 4).

Of importance, arginase inhibition reduces the production of interleukin-8 in vascular endothelial and smooth muscle cells as a reaction to native low-density lipoprotein (LDL) [52]. Moreover, the mitochondrial membrane potential (MMP), which is generally decreased upon native LDL stimulation, is restored upon arginase inhibition. Thus, inhibition of arginase has been proposed to treat a list of cardiovascular diseases [53].

Growing evidence suggests a role that arginase plays in the pathogenesis of neurodegenerative diseases [54]. The activity and expression levels of arginase are significantly higher in the hippocampi of AD patients and AD mice [11, 55, 56]. Moreover, substantially reduced levels of arginase substrate, arginine, are observed in the cortices of AD patients [57]. The intriguing question of how Arg1 and Arg2 differ in their biological function and regulation under various pathological conditions remains an area of intensive research.

## Ornithine decarboxylase

ODC is the rate-limiting enzyme for polyamine synthesis that decarboxylates ornithine to form putrescine (Figure 2). The adult brain contains substantial amounts of ODC protein, but its enzymatic activity is relatively low in the healthy adult brain. In contrast to other metabolic pathways, a family of specific antizymes inhibits ODC, and regulates polyamine transport [58]. ODC antizyme (OAZ) is a natural ODC inhibitor (Figure 2). Its binding to ODC also causes fast degradation of the enzyme [59].

Remarkably, OAZ is firmly preserved over evolution; however, its detailed function is poorly understood. The OAZ expression is promoted by high cellular polyamines’ concentration, perhaps as a feedback mechanism to limit polyamine modulation of N-methyl-D-aspartate receptors (NMDAR), and the adult brain shows a relatively high expression of OAZ [60]. In the brain, the antizyme typically co-localizes with NMDARs of the cortical pyramidal cells [61], which indicates its role in regulating channel functions.

Of note, a substantial accretion of OAZ in the brains of AD patients compared to healthy individuals has been reported. Remarkably, in these cases, OAZ preferentially accumulates in the neuronal bodies and axons of the hippocampus. Thus, it was proposed that AD-related OAZ accumulation possesses neuroprotective functions [61]. It is plausible to hypothesize that the upsurge in antizyme levels in the AD brains reflects the natural mechanism of reduction the polyamine pathway overactivation-associated toxicity, as well as NMDAR modulation, which is inadequate in the conditions of chronic PSR.

Of importance, AD-associated ODC translocation from nucleus to cytoplasm in the pyramidal cortical cells has been reported [62]. Nilsson et al*.* elegantly evidenced an early shift of the ODC immunoreactivity from the nuclear compartment towards the cytoplasm in AD brains. This pattern of expression resembles the mentioned above translocation of Arg2 to the cytoplasm. Accordingly, we suggest that these characteristic translocation phenomena indicate AD-related changes in polyamine metabolism dependent upon subcellular localization of its principal enzymes (Figure 4).

## Polyamine catabolism

Spermidine/spermine acetyltransferase (SSAT) is the rate-limiting polyamine catabolism enzyme that acetylates polyamines and converts them to functionally inactive forms via alteration in charge (Figure 2) [63]. Additionally, spermidine and spermine acetylation facilitate their degradation and excretion. Many factors are capable of inducing the polyamine catabolism. Polyamines, corticosteroids, estradiol, growth hormone, non-steroidal anti-inflammatory drugs instigate SSAT levels and activity [64].

Polyamine oxidase (PAO) oxidizes acetylspermine and acetylspermidine to form spermidine and putrescine respectively, together with aminopropanal and hydrogen peroxide. Additionally, spermine is oxidized by spermine oxidase (SMO) to produce hydrogen peroxide, and acrolein (Figure 2) that exert numerous cytotoxic, mutagenic, and immunosuppressive effects [65, 66].

Remarkably, acrolein levels are significantly increased in AD patients’ hippocampi [67]. Additionally, acrolein adducts are present in dystrophic neurites surrounding senile plaques [68]. Accordingly, acrolein’s role in the AD pathogenesis has been suggested [69]. Moreover, proved neurotoxicity of the polyamine degradation products such as hydrogen peroxide, acrolein, and aminopropanal, led to the "aldehyde load" hypothesis of neurodegenerative disorders [70, 71].

## General biological functions of polyamines (with focus on CNS)

We have mentioned that polyamines carry positively charged nitrogen moieties. DNA is typically negatively charged polymer due to the presence of negatively charged phosphate ions in the sugar-phosphate backbone. In his pioneering work, Tsuboi Masamichi (1964) reported that the distances between the charged entities of polyamines correspond well to the distances between DNA phosphates [72]. The author proposed an original model of spermine interaction with the DNA molecule, which leads to stabilization of its double-helical conformation. More recent studies proved that large amounts of the intracellular polyamines are associated with DNA and RNA and capable of influencing the genes’ transcription and translation rate [73, 74]. Muscari et al*.* demonstrated that spermine efficiently bounds to the DNA strands and exerts a potent antioxidative effect at physiological (0.1 mM) concentration [75]. Spermine has also been shown to scavenge free radicals and protect DNA from the ROS-associated damage [76].

Remarkably, polyamines demonstrate dissimilar effects on gene expression in relation to their concentration. They enhance gene expression at low concentrations but completely inhibit at high concentrations [77]. Of note, the polyamines concentrations used in the study [77] are at physiological range (0.1-2mM). The authors speculate that polyamines provide favorable conditions for RNA polymerase to access DNA segments with a reduced negative charge. A similar pattern of concentration-dependent differential effects of polyamines on the initiation and elongation of protein synthesis has been reported by Giannakouros et al*.* in an original experiment in cell-free system [78]. Accordingly, a dual role of polyamines in stress is related to their ability to turn on some stress-responsive genes but shut down other genes has been suggested [1].

In relation to the CNS, polyamines show numerous physiological effects, which support normal neuronal function and axonal integrity. At functional level, polyamines modulate complex cognitive processes and facilitate associative memory acquisition and recall [79] [80]. However, besides regulation of basal cellular functions, in the mammal brain polyamines subserve highly specific tasks. In neurons, they regulate gating of several ion channels. In some cases, the mechanism is very simple. Membrane depolarization attracts cytosolic polyamines into the channel pore, which prevents ion flow [81] (Figure 3). This mechanism may moderate overexcitation associated with acute trauma. Additionally, polyamines have been shown to confer inward rectification to certain potassium channels, α-amino-3-hydroxy-5-methyl-4-isoxazolepropionic acid (AMPA) receptors, and nicotinic acetylcholine receptors (nAChR). Intracellular spermine, particularly, is responsible for intrinsic gating and rectification of inward rectifier potassium channels by plugging the channel pore [82]. Moreover, polyamines bind with high affinity to the omnipresent inwardly rectifying potassium channels. This process efficiently facilitates influx of potassium and affects electrolyte balance, resting membrane potential, and the cell electrical activity [83] (Figure 3).

Pioneering studies by Williams et al*.* (1989) suggested that NMDA receptor complex possesses a polyamine recognition site [84]. Considering the NMDA receptor as the predominant molecular device for controlling synaptic plasticity and memory function at cellular level, polyamines appear to efficiently modulate human memory. Later works demonstrated multiple effects of extracellular spermine and evidenced polyamine binding to glutamate-sensitive NMDA, AMPA, and kainite receptors [82, 85].

Considering the complex effects of polyamines upon the neuronal activity via interaction with numerous ion channels it is explicable why the polyamines pathway is highly responsive to pathological brain conditions like trauma [7], stroke [86] and epilepsy [87], and plays a role in the pathogenesis of numerous mental disorders. Of note, substantial alterations in the levels of polyamines have been demonstrated in schizophrenia, mood disorders, anxiety and suicidal behavior [88, 89]. Though, the direct causal relationship between the events is still debated in the literature.

Of importance, various pathophysiological processes associated with neuronal damage have been shown to induce activation of putrescine synthesis. Dempsey et al*.* utilized bilateral carotid artery occlusion to produce dense forebrain ischemia and demonstrated enhanced levels of ODC in cortical neurons of ischemic gerbils [90]. However, in this case, the authors propose that ODC is a causative factor or, at least, a marker, which is associated with metabolic events leading to progressive functional deterioration after cerebral ischemia. Paschen et al*.* used hypoglycemic coma in rats to induce significantly increased putrescine levels throughout the brain [91]. The authors speculate that the increase in putrescine content is an early marker of neuronal cell necrosis regardless of the injury pathogenesis.

Sauer et al*.* occluded cerebral artery to induce ischemia in rats [92]. The procedure led to elevation in the levels of putrescine in infarcted and non-infarcted areas. Remarkably, treatment with difluoromethylornithine (DFMO), an inhibitor of ODC, prevented the ischemia-induced increase in putrescine levels; though, it did not affect the infarct volume, which indicated polyamines function as protective rather than pathogenic. Subsequently, it has been proven in numerous models that the universally observed after acute neuronal damage ODC activation followed by putrescine accumulation are the means of cellular protective mechanisms [93–95].

Gilad and Gilad (1992) pointed out that the capability of polyamines to modulate the ion channels functions may assume importance in cellular defense mechanisms [7]. The authors revolutionarily proposed that neurotrauma-related induction of the inherently transient polyamine response is an integral part of protective biochemical programs that are vital for neuronal survival. Consequently, the authors further developed their model and suggested that the brain PSR, is a common reaction to stressful stimuli, including physical, emotional, and other stressors, with a magnitude directly correlated with the stress intensity [17]. The researchers indicated that traumatic injury of extreme degree results in an incomplete PSR associated with accumulation of putrescine but reduction in the levels of the higher polyamines, spermidine and spermine.

This model explains the protective effects of the systemic polyamines application evident in forebrain neurons following acute ischemia [96]. Direct application of polyamines has also been shown to be neuroprotective. For instance, L-arginyl-3,4-spermidine neuroprotective properties have been proven in several *in vitro* models of neurodegeneration and *in vivo* transient forebrain ischemia rat model [97].

Extracellularly applied arginase has been proven to be a potent neuroprotector and a nitric oxide-independent inhibitor of neuronal apoptosis [41]. Moreover, upregulation of Arg1 leading to a substantial upsurge in spermidine synthesis has been shown to promote axonal regeneration [98]. Accordingly, we suggest that Arg1 is an integral part of the brain adaptive PSR.

Recently, Dhara et al*.* elegantly demonstrated that polyamines control assembly of neuronal nicotinic α4β2 and α7 acetylcholine receptors [99]. This capability is unique, since polyamines do not modulate assembly of any other ion channels. Remarkably, lowering polyamine levels upregulates brain α4β2 and α7 levels. The authors evidence strong correlation between increased acetylcholine-evoked currents and SSAT activity. Additionally, they show that SSAT promotes cell-surface expression and assembly of nAChRs by catalyzing polyamines. Strikingly, DFMO pretreatment leads to the same phenotype [99].

Of importance, α4β2 and α7AChR are the most abundant nAChR in the brain that control various aspects of synaptic signaling and plasticity related to memory acquisition and recall [100]. It seems that polyamine control on their function possesses evolutionary significance. We suggest that acute PSR, caused by trauma, for instance, leads to a transient reduction in the density of nAChRs, which protects the brain against excitotoxic damage. In contrast, AD-associated chronic PSR is followed by a persistent and substantial loss of nAChRs, which is partially responsible for the development of cognitive decline [100]. Considering pathogenic significance of α7AChR in AD, the described polyamine aptitude to control the receptor assembly may explain some features of this devastating disease and be tailored for its treatment.

In order to investigate the precise function of polyamines, numerous models have been utilized. Halmekyto et al*.* (1991) generated transgenic mice carrying intact human ODC gene and demonstrated expression of human-specific ODC mRNA across tissues [101]. In contrast to their wild-type littermates, the transgenic mice exhibited a significantly elevated enzyme activity. Of note, ODC activity was moderately elevated in parenchymal organs such as the liver, kidney, and spleen of the transgenic animals. However, the most remarkable difference has been found in the brain tissue, where the ODC activity was about 70 times higher.

It is worth mentioning that the extremely elevated ODC activity was not followed by changes in the polyamines’ content. The only change in most tissues was an increase in the spermidine to spermine ratio. However, testis and brain demonstrated a different pattern with a dramatic upsurge in putrescine levels. Of note, the elevated brain ODC activity and accumulation of putrescine in the transgenic animals did not result in morphological changes [101].

To clarify whether the high putrescine content influences the functional properties of the brain tissue, Halonen et al*.* took advantage of the same model overexpressing the human ODC gene and performed behavioral tests [93]. The authors evaluated the rate of memory acquisition in a maze and showed that transgenic mice had significantly impaired performances. Additionally, they proved the aberrant expression of the transgene was most prominent in the brain and led to dramatic increase in putrescine levels. Moreover, the seizure threshold to chemical and electrical stimuli was significantly elevated, even though the concentrations of glutamate and GABA were not changed. Subsequently, the authors suggest that the observed increase in ODC activity following by escalation in brain putrescine levels is neuroprotective rather than detrimental [93].

Polyamines’ supplement has been shown to be beneficial for mental health and improve memory acquisition and recall in humans and rodent models [102]. However, putrescine application leads to neurotoxicity in some circumstances in rodents. Several lines of evidence prove that the systemic administration of putrescine in supraphysiological doses induces a characteristic toxic response. De Vera et al*.* (1992) injected rats with putrescine 200 mg/kg, which consequently displayed a behavioral pattern that included wet dog shakes and motor discoordination [103]. Of note, the brain concentration of putrescine correlated with the severity of clinical signs. Moreover, histological examination proved the presence of perivascular edema and spongiosis, which were present two hours after the treatment. The authors indicated that the putrescine effects are similar to those of kainic acid at convulsant doses.

Several studies utilized models with local brain administration of polyamines. De Sarro et al*.* injected putrescine directly into the rats’ pre-piriform cortex [104]. The procedure elicited bilateral clonic seizures. Remarkably, injection at the same site of the selective NMDA receptor antagonist prior to putrescine, prevented the seizures’ development. Moreover, injection of dizocilpine, a specific inhibitor of the polyamine site at the NMDA receptor, before putrescine, significantly protected against seizures elicited by this polyamine.

Sparapani et al*.* utilized an *in vitro* neuronal system, consisting of primary rat cerebellar granule cells, to study the neurotoxicity of spermine, spermidine, and putrescine [105]. The mature cultures exposed to increased concentrations of spermine showed dose-dependent cells’ death, with the half-maximal effect below 50 μM. Spermidine demonstrated toxicity, which was about 50% that of spermine; and putrescine showed a moderate toxicity. Of note, spermine-caused neuronal death was apoptotic by nature and has been prevented by application of the NMDA receptor antagonists. The authors speculate that polyamines are toxic to granule cells and their toxicity is mediated by the NMDA receptors.

De Vera et al*.* (2008) investigated the effects of spermine and putrescine in human primary cerebral cortical cultures containing both neurons and glia [106]. Both spermine and glutamate were toxic to aged neurons (cultures of 26^th^ and later division), though putrescine induced relatively minor effects. Remarkably, spermine toxicity was inhibited by both Dizocilpine and Ifenprodil, which points to an NMDA receptor depending mechanism.

Several groups demonstrated that numerous pathological factors, including toxic and mechanical lesions, are capable of inducing ODC activity following by a rapid but transient elevation in the brain putrescine levels [107–111]. It seems that various stimuli elicit a typical PSR in the brain and the magnitude of the response is stimuli-dependent.

## Crosstalk between S6K1 and Arg2

The mechanistic target of rapamycin (mTOR) is a kinase encoded by the MTOR gene [112]. mTOR serves as a central component of two distinct protein complexes, mTOR complex1 and complex2, which regulate essential cellular processes, and function as serine/threonine kinases to control cell growth, proliferation, motility, and survival [113]. mTOR signaling has been shown to be overactive in AD brains and contribute to disease progression [114].

The ribosomal protein S6 kinase beta-1 (S6K1) is a downstream target of mTOR. Mounting evidence indicates an interesting phenomenon related to interaction between S6K1 and Arg2 [115]. This interaction may be involved in degeneration and aging. Of note, S6K1 levels and activity are resolutely increased in various human aging tissues, which may play a causal role in age-associated NOS-uncoupling, oxidative stress, and senescence. Remarkably, S6K1 overexpression upregulates Arg2 expression and contrariwise, S6K1 silencing in senescent cells reduces Arg2 expression [115]. Moreover, inhibition of S6K1 in the senescent cells decreases Arg2 expression and activity, pointing to a regulatory role of S6K1 activity in Arg2 function and *vice versa;* Arg2 gene silencing in senescent endothelial cells has been shown to reduce S6K1 activity. On the other hand, Arg2 knockout eliminates S6K1 overactivity [115]. Remarkably, Arg2 deficiency extends lifespan in mice [116]. This phenomenon is in the line with studies evidencing an antiaging effect of mTOR inhibitors, particularly rapamycin [117].

Several reports link mTOR signaling alterations to age-associated cognitive decline and AD pathogenesis pointing to this kinase as the crossroad between cognitive aging and AD [118, 119]. Remarkably, inhibition of mTOR with rapamycin abolishes cognitive deficits and diminishes Aβ levels in a mouse model of AD [120]. Moreover, reducing S6K1 expression improves spatial memory and synaptic plasticity [121].

Of note, mTOR complex1 negatively regulates autophagy, which is the indispensable cellular mechanism to eliminate unnecessary and dysfunctional components [122]. The housekeeping role of autophagy in the brain is particularly evident in neurons loaded with pathogenic misfolded proteins, such as Aβ aggregates [123]. In AD the autophagic-lysosomal dysfunction causes severe neurodegenerative phenotypes associated with accumulations of lysosomes and autophagic vacuoles (Figure 4) [124, 125].

Additional evidence indicates that impaired autophagy and enhanced Arg2-mTOR crosstalk are strongly implicated in vascular aging and atherosclerosis. Xiong et al*.* credibly demonstrated that Arg2 impairs endothelial autophagy function independently of the arginine ureahydrolase activity but via activation of S6K1 [126]. Accordingly, it was hypothesized that disruption of the S6K1-Arg2 crosstalk by inhibition of Arg2 or S6K1 may restore NOS function, improve NO production, reduce inflammation, and eventually, preclude senescence and decelerate aging [127].

More recent investigations have deciphered the precise mechanisms of the mTOR complex1 pathway Arg2-associated activation contributing to cell senescence and apoptosis. It was shown that overexpression of Arg2 induces the re-distribution of lysosome and mTOR from perinuclear area to cell periphery and activation of mTOR-S6K1 pathway (Figure 4) [128]. Therefore, S6K1-Arg2 crosstalk represents a promising therapeutic target to slowdown the age-associated processes and treat the neurodegenerative diseases.

## Evolutionary perspective on aging and Alzheimer's disease

Inevitable aging and death are universal phenomena across multicellular organisms, indicating that they are the natural consequences of life and are not related to disease. Moreover, average lifespan is a species-specific characteristic, which varies significantly between different species pointing to natural limits of life expectancy. Nevertheless, in recent decades, human longevity well exceeds the ever-chronicled numbers. Still, this spectacular phenomenon is accompanied by increased somatic and mental morbidity. In this context, AD is a very interesting example of the evolution-related pathology. AD is a peculiar and specific to *Homo sapiens* pathology that may relate to adaptive changes transpired only in evolutionary recent history. Apparently, many of the AD-associated risk-factors were not present prior to industrialization era, potentially reducing the prevalence of the disease in ancient times. However, the recent theories of aging predicated upon evolutionary concepts provide an alternative explanation for the recently increased AD morbidity [129].

In accordance with a prevailing view, the senile dementia of the Alzheimer type is a logical consequence of continuously increasing lifespan and an inevitable manifestation of senescence. Some estimations even predict dementia by the age of 130 for everyone as an unescapable toll of longevity [130]. Another hypothesis of “antagonistic pleiotropy” has been articulated by several groups who comprehend some genes’ expression to be extremely beneficial or even necessary during an early phase of the life cycle but detrimental at the late phase [131].

This approach refers to the incredible plasticity of the human brain, which is critical for learning and memory during development, as a particularly beneficial capability associated with an increased expression of neuroplasticity-related genes. On the other hand, this theory considers these genes’ expression as a detrimental and bioenergetically costly event for the aging brain, which plays a critical role in the AD pathogenesis [132]. Accordingly, it was hypothesized that susceptibility genes associated with AD development possess pleiotropic effects [133]. The ubiquitous in ancestral species APOEε4 precursor gene has become the strongest AD genetic risk-factor [134]. However, this ε4 allele is apparently beneficial for young individuals’ mental and physical health and confers a risk for atherosclerosis and cognitive decline only in advanced ages. Hence, it was proposed that APOE gene represents an example of antagonistic pleiotropy [135]. Antagonistic pleiotropy has also been suggested for *tau*-protein role in the AD development [136].

Mounting evidence indicates general down-regulation of polyamine biosynthesis during aging [137]. Pioneering work by Duffy and Kremzner (1977) in human fibroblasts demonstrated that cell senescence is associated with the reduction in ODC activity [138]. Beyer et al*.* established that aging alters ODC activity and leads to decrease in polyamine content [139]. Lin et al*.* reported a significant 2.65-fold down-regulation of ODC levels and 4.73-fold of PAO levels in 24-month-old mice muscle tissue compared to 3-month-old mice [140]. Accordingly, spermidine intracellular concentrations tend to decline during normal aging as well; though, its administration evidently extends the lifespan in various model organisms, including yeast, worms, flies, and mice [141]. Contrariwise, depletion of endogenous polyamines causes hyperacetylation, ROS generation, and early necrotic death eventuating in diminished lifespan [141].

Surprisingly, Arg1 levels gradually increase in skeletal muscles of mice during aging [142], nevertheless, its upturn is not followed by elevation in polyamine levels. This phenomenon may reflect a compensatory mechanism, which in any case is inadequate to support the polyamine content in aged organism, and causes arginine deficiency, NOS and ADC substrate deprivation. Of importance, the age-associated upregulation of arginase has been suggested to be involved in various pathologies. Endothelial dysfunction [143], hypertension [144], and diabetes mellitus [145] are among them.

In this context, we comprehend aging as an extremely intricate genetically programmed and epigenetically influenced natural biological process, which may be accelerated by stressful or harmful stimuli leading to cell senescence and eventually death.

## Polyamine pathway in Alzheimer’s disease

Despite a century-long investigation, no clear understanding of AD etiology and pathogenesis is achieved. Several definite hallmarks of the disease have been described and studied with precision, however, the causal relationships between them and clinical dementia remain to be revealed. Mounting evidence indicates that AD is a pervasive metabolic disorder characterized and possibly caused by dysregulation of numerous biochemical pathways, which underlie its complex pathogenesis [146, 147].

Many groups have tried to correlate the AD-associated brain metabolic changes with clinical manifestation and objective morphological alterations. In their pioneering work, Bernstein and Müller (1995) convincingly demonstrated augmented immunopositivity for ODC in neocortical neurons of AD patients [148]. Accordingly, the authors hypothesized that the polyamine system is actively involved in neurorestorative processes taking place in AD brains trying to cope with emerging neurodegeneration. Additionally, they proposed that ODC is activated to modulate the NMDA receptor function. Morrison and Kish proved that brain polyamine levels are substantially altered in AD brains [149]. The authors suggested that abnormal polyamine metabolism is involved in the AD-associated neurodegeneration due to its influence upon calcium dynamics and glutamate receptors function. The scientists substantiated the early rodent-based data by human postmortem research and demonstrated significantly elevated brain levels of ODC in the perinatal period, which indicated a developmental role of polyamines [150]. Moreover, the authors linked the increased levels of ODC in the temporal cortex of AD patients’ brains with the disease progression.

Yatin et al*.* confirmed in an elegant *in vitro* study that Aβ-treated hippocampal neurons show an increased polyamine metabolism in response to free radical-mediated oxidative stress [151]. Additionally, the authors showed that the free radical scavenger, vitamin E, application prevents these attenuations. Thus, the authors speculated that the observed polyamine response is a reaction to Aβ-mediated oxidative stress. However, in a modified study, the same group demonstrated a strong synergistic neurotoxic effect of Aβ applied together with spermine to treat cultured neurons [152]. The authors suggest that Aβ-related spermine accumulation is harmful to neurons and hypothesize that in the AD brains polyamine pathway’ regulatory enzymes are damaged by oxidative insults and incapable of polyamine synthesis and uptake’s regulation, which leads to accumulation of intracellular polyamines up to toxic levels. This hypothesis accords with the data acquired in the pioneering *in vivo* experiments utilizing an ODC inhibitor, DFMO, in the brain ischemia/reperfusion paradigm in rodents [153]. It has been well-established that administration of DFMO results in a dose-dependent beneficial effect upon hippocampal neurons survival rate indicating that ODC activity and the polyamines’ levels play a significant role in the brain response to ischemic injury.

The same ODC inhibitor has been used by Kan et al*.* to show protective effect of the treatment on cognitive functions in AD mice [8]. The authors disclosed a substantial cerebral arginine deprivation and immune suppression caused by arginase overexpression in the AD mice brains. Despite a very promising phenotype observed in AD model, subsequent 12-month-long clinical trial did not demonstrate a noticeable effect in an AD patient [154].

Rodent studies prove a substantial polyamine dysregulation in a model of tauopathy [155]. Accordingly, it has been proposed that pathological *tau* represents a chronic physiological stressor provoking a typical PSR. The authors took advantage of transgenic mice harboring human *tau* P301L associated with frontotemporal dementia mutation to show the brain accumulation of putrescine and acetylated spermidine. Additionally, they demonstrated that acetylspermidine accretion intensifies *tau* oligomerization, however, SSAT repression reduces *tau* aggregation. These data signify a detrimental role of polyamine acetylated products accumulation on *tau* fate in the brain [155].

Of importance, several sets of data pointed to arginase, which is up-stream to ODC enzyme, as the main cause of the AD-related polyamine pathway abnormalities. Hansmannel et al*.* revealed a significant escalation of Arg2 levels in AD brains compared to healthy controls [156]. Moreover, the authors associated the presence of the rare Arg2 allele with increased risk of AD onset and posed a question on the urea cycle involvement in AD pathogenesis. Additionally, in the mentioned above murine study [8] the researchers revealed a significant elevation in the brain Arg1 levels correlating with the onset of cognitive decline. We published data showing a significant increase in the intracellular Arg1 levels in the hippocampal neurons of AD mice compared to wild-type animals [11]. A pioneering metabolomic human study revealed dramatically elevated (by several folds) levels of urea, a by-product of arginase (Figure 2), in all AD patients’ brain regions compared to healthy controls indicating disease-associated overactivation of arginase [157].

Other groups utilized advanced techniques to prove the AD-associated polyamine metabolism disturbances. Inoue et al*.* applied ultra-performance liquid chromatography coupled with mass spectrometry to profile and differentiate metabolically the frontal, parietal, and occipital lobes of the AD patients’ brains in comparison with healthy controls [9]. The authors disclosed a significant increase in spermidine, spermine, and putrescine levels, without a change in ornithine levels in frontal and parietal lobes of AD patients’ brains.

Liu et al*.* compared the metabolic profile of arginine and its downstream metabolites in brains from AD patients with healthy brains to reveal significant differences [158]. The authors also analyzed the activity and protein levels of NOS and arginase and demonstrated their inverse age- and region-specific alterations linked to significant elevation in the rate of arginase activity.

Recently, Mahajan et al*.* (2020) applied a targeted metabolomic and transcriptomic study to demonstrate dysregulation of multiple metabolic networks related to brain transmethylation and polyamine pathway in AD [159]. The authors reported significant, correlating with severity of disease, alterations in concentrations of numerous metabolites in AD brains compared to control samples. These metabolites represent biochemical reactions in the polyamine pathway (with significantly higher spermidine concentrations in AD brains) and urea cycle. A transcriptomics analysis accords with the metabolomics results, further revealing significant alterations in gene expression of pivotal polyamine metabolism regulators. Of note, SSAT and PAO demonstrated significantly elevated levels of expression in entorhinal cortex and hippocampus of AD patients’ brains.

Remarkably, the levels of ornithine are generally normal in AD brains despite a significant elevation in the urea levels, which is a product of the same reaction (Figure 2), even though the levels of arginine and its brain bioavailability are decreased [57]. This paradox misled some researchers to speculate that the defective urea clearance characterizes AD [157]. Though, apparently, ornithine generated by up-regulated arginase in AD brains is immediately consumed by OTC and ODC, which are overactivated as well [54].

It is noteworthy that metabolomics investigations of AD patients’ CSF detect a relative disease-associated reduction in arginine levels [160, 161]. Moreover, the patients with mild cognitive impairment (MCI) are characterized by substantially lower than in controls urine arginine levels [162]. These patients also demonstrate a reduced global arginine bioavailability ratio positively correlating with the Mini-Mental Status Examination score, which points to diminished urinary arginine levels as an early diagnostic biomarker of MCI and AD.

Additionally, metabolomics studies of human plasma clearly indicate differentially affected polyamine and arginine metabolism in AD patients. As a result, the subjects with MCI are easily distinguishable from healthy controls and AD patients [163], which gives hope for AD diagnosis at early stages with routine laboratory blood tests and further supports our perspective on AD as a brain expression of a complex metabolic disorder [146].

It should be pointed out that arginase upregulation in the AD brain causes NOS and ADC substrate deficiency. In this case, NOS generates diminished amounts of NO and switches into production of superoxide anion, which aggravates the oxidative stress (Figure 4B). Additionally, ADC lacking the regular substrate, arginine, consumes ornithine to produce putrescine, which further upsurges the polyamines’ levels (Figure 2). Moreover, spermine directly inhibits NOS activity in various cell types. In the rodent brain this effect upon NOS1 activity is very prominent [164] and leads to improvement in arginine bioavailability for arginase in the AD brain, facilitates the polyamine synthesis, and closes the vicious circle of neurodegeneration.

On the other hand, overactivated PAO generates hydrogen peroxide to exacerbate the oxidative damage (Figure 4B). Likewise, a relative decline in the levels of agmatine deprives the brain of the potent neuronal protector [165].

In our studies, we utilized a non-competitive arginase inhibitor, norvaline, which also inhibits S6K1 [166], to moderate the activity of the brain arginase in AD mice [11]. The spatial memory was significantly improved in the treated animals, and the improvement was associated with a reduction in neuroinflammation. Additionally, we evidenced a treatment-related decline in β-amyloidosis [167], followed by an improvement in the BBB integrity [168] and neurogenesis [169]. Of note, the hippocampal levels of Arg1 protein significantly reduced following the treatment. Yang et al*.* credibly demonstrated that Arg1 expression is regulated by histone deacetylase 4 (HDAC4)-mediated histone acetylation [170]. Our data proved that HDAC4 levels decrease dramatically following the treatment with norvaline. Moreover, its functionally active form, demonstrated a substantial reduction in levels [169]. Of importance, HDAC inhibitors are promising AD-modifying agents [171], therefore, we speculate that HDAC is partially responsible for the phenotype we observed.

Our approach has been recently tested with another non-competitive arginase inhibitor, citrulline, in AD mice [172]. The treated animals demonstrated increased arginine CSF levels and performed significantly better in a maze. The authors demonstrated that the therapeutic time window is limited to the period prior to the development of cognitive symptoms, like in our studies. Consequently, we suggest that arginase gradually alters its functional role during the disease progression and propose that treatment strategy directed at fine-tuning of arginase activity with natural inhibitors is a promising AD-modifying approach capable of interfering with various aspects of this pathology. Moreover, we state that polyamine metabolism dysregulation is a causal factor of neurodegeneration, but not just a signature. Still, regardless of several successful attempts to interfere with PSR activity at various levels (Table 1) and halt the development of AD-like pathology in animal models, the chicken or the egg causality dilemma remains to be resolved.

**Table 1:** Possible theranostic applications and their effects within the polyamine metabolism.

**Target gene**	**Manipulation/ agent**	**Mechanism**	**Effect**	**References**
ODC	DFMO	Irreversible ODC inhibition	Neuroprotective	[8]
SSAT	KO mouse	Deletion of SSAT	Neuroprotective	[155]
PAO	MDL 72527	Irreversible PAO inhibition	Neuroprotective	[180, 181]
SMO	Transgenic mouse	SMO overexpression	Neurotoxic	[182]
Arg1, Arg2	Norvaline	Noncompetitive arginase inhibition	Neuroprotective	[11, 167–169]
Arg1, Arg2	Resveratrol	Noncompetitive arginase inhibition	Neuroprotective	[183]
Arg1, Arg2	Chloroquine	Competitive arginase inhibition	Neuroprotective	[184]
Arg1, Arg2	Citrulline	Noncompetitive arginase inhibition	Neuroprotective	[172, 185]
Arg2	KO mouse	Deletion of Arg2	Neuroprotective	[186]
Arg1	AAV-mediated overexpression	Arg1 overexpression in the hippocampus	Neuroprotective	[173]
Arg1 in myeloid cells	Transgenic mouse	Arg1 haploinsufficiency	Neurotoxic	[174]

In this context, it is worth noting that several groups tried to manipulate Arg1 expression in the models of familial frontotemporal dementia [173] and AD [174]. Remarkably, Arg1 overexpression mitigated hippocampal atrophy in transgenic mice, but Arg1 deletion in myeloid cells increased *tau* accumulation relative to Arg1-sufficient mice [173]. Of note, rTg4510 mice used in this study express a human *tau* containing the mutation linked to familial frontotemporal dementia. The animals progressively develop age-related neurofibrillary tangles, neuronal loss, and behavioral impairments [175]. The tangles are observed by about 4 months of age and the deficits in spatial navigation are seen as early as 1.3 months of age [175]. In the cited above study [173], four-month-old rTg4510 mice were injected in the hippocampus with an rAAV9-Arg1 construct. The animals demonstrated elevated levels of Arg1 throughout the hippocampus that was associated with reduced tangle pathology; however, no behavioral effect has been reported. Another recent study utilized haploinsufficiency of Arg1 in myeloid cells of Tg2576 mice [174]. This manipulation promoted Aβ deposition and exacerbated behavioral impairment.

There are several possible explanations for these contradictory results. First of all, overexpression methodology obviously does not represent physiologic phenomena [176]. It creates supraphysiological levels of protein that dysregulate many biological pathways, interfere with the protein assembly, which severely confuses the results’ interpretation [177]. Knockout technique is problematic and sometime inappropriate as well [178], because, apparently, the absence of one gene alters expression of other genes and changes entire developmental programs [179]. Nevertheless, the question of how Arg1 and Arg2 differ in their biological function is still open.

Furthermore, in our opinion, it is critical to start the treatment prior to any cognitive impairment symptoms, literally precluding the neurodegeneration. This is the only way to eradicate dementia. There are sensitive periods for the most successful therapeutic intervention. After the vicious cycle is already ongoing and neurodegeneration is pronounced, it is too late to intervene, unfortunately.

## CONCLUSIONS

For decades the confirmed diagnosis of AD had been dependent upon the postmortem brain investigation and analysis of the β-amyloid deposition patterns and identification of the neurofibrillary tangles. Nevertheless, the canonical pathological changes poorly correlate with clinical manifestation, laboratory findings, and even the prognosis of the disease. Moreover, a cornucopia of clinical trials aimed at lessening of amyloid and/or *tau* brain burden, yielded no reliable disease-modifying therapy. Thus, it seems that the presence of amyloid plaques and neurofibrillary tangles in the brain does not presume the causal relationships between the hallmarks and other AD-associated neurodegenerative processes and cannot be recognized as etiological factors.

A novel perspective on AD, with emphasis upon its evolutionary and metabolic aspects, proposes an inclusive reassessment of the causal relationships between traditional hallmarks, homeostatic features, and clinical manifestations. Accordingly, we comprehend AD as a chronic disease characterized by an unwinding vicious cycle of metabolic aberrations with a series of pathogenetic steps that reinforce each other, lead to neurodegeneration, and eventuate in clinical dementia. We envision the AD-associated polyamine response as an integrated part of the conserved adaptive mechanism and emphasize that prolonged induction of polyamines possesses limited efficacy in coping with gradual oxidative stress and may not have beneficial effects due to toxicity issues. We also underline that continual induction of the polyamine pathway is followed by arginine brain deprivation, extensive catabolic oxidation of polyamines, ROS generation, and induction of oxidative stress, which aggravate the AD symptoms.

Accruing evidence highlights polyamines as the pivotal players in signaling responses to various environmental stimuli, which are involved in various aspects of metabolism, maintenance of antioxidant capacity, and osmotic regulation. This pathway represents a metabolic hub existing virtually in all phyla, including simple organisms, plants, and mammals, and constitutes an evolutionary-conserved adaptive response.

We indicate the beneficial role of the polyamines’ levels elevation following a short-term stimulus. While chronic stress, in some conditions, and in aging organism principally, may lead to an aberrant polyamine metabolism, which becomes maladaptive. Enduring stimuli such as repetitive brain trauma, cerebral arteriosclerosis-associated ischemia, metabolic stress, *etc.* lead to a deviant PSR and initiate the vicious cycle of neurodegeneration with distinctive β-amyloid aggregation and *tau* protein hyperphosphorylation, the main hallmarks of AD, which are just epiphenomena of the upstream pathology. Accordingly, we propose fine-tuning of arginase activity with natural inhibitors to halt the development of AD-associated cognitive decline.

Additionally, we suggest that AD may be driven by various pleiotropic mechanisms, which deserve close attention and further research. This view approaches the enigmatic AD pathogenesis within the framework of evolutionary sciences that comprehend some genes as necessary for early development, but which are harmful in the elderly. Consequently, we believe that investigation of the fragile equilibrium between neurodegenerative and neuroprotective effects of ROS, NO, arginase, β-amyloid, APOEε4, and intimately pertinent to them, neuroinflammation in the aging brain, may eventually decipher the AD pathogenesis conundrum and lead to efficient disease-modifying treatment.
